# On the Z_1_-dependence of electronic stopping in TiN

**DOI:** 10.1038/s41598-018-36765-7

**Published:** 2019-01-17

**Authors:** Mauricio A. Sortica, Valentina Paneta, Barbara Bruckner, Svenja Lohmann, Tomas Nyberg, Peter Bauer, Daniel Primetzhofer

**Affiliations:** 1 Department of Physics and Astronomy, Uppsala University, Box 516, S-751 20 Uppsala, Sweden; 2 Atomic Physics and Surface, Johannes Kepler University, A-4040 Linz, Austria; 3 Department of Engineering Sciences, Uppsala University, Box 534, S-751 21 Uppsala, Sweden

## Abstract

We present a thorough experimental study of electronic stopping of H, He, B, N, Ne and Al ions in TiN with the aim to learn about the energy loss mechanisms of slow ions. The energy loss was measured by means of time-of-flight medium-energy ion scattering. Thin films of TiN on silicon with a δ-layer of W at the TiN/Si interface were used as targets. We compare our results to non-linear density functional theory calculations, examining electron-hole pair excitations by screened ions in a free electron gas in the static limit, with a density equivalent to the expected value for TiN. These calculations predict oscillations in the electronic stopping power for increasing atomic number Z_1_ of the projectile. An increasing discrepancy between our experimental results and predictions by theory for increasing Z_1_ was observed. This observation can be attributed to contributions from energy loss channels different from electron-hole pair excitation in binary Coulomb collisions.

## Introduction

When an energetic ion moves in matter, it is decelerated due to interaction with the target electrons and nuclei; interactions which are known as electronic and nuclear stopping, respectively. The mechanisms involved in these energy transfer processes are of great interest in many research fields as e.g. ion implantation^1^, medicine^2^ and materials science^3^. The mean energy loss of the ion per unit path length when travelling in a material is usually expressed by the force the medium exerts on the ion, known as the stopping power, *S*, or more conveniently by the stopping cross section (SCS), *ε* = *S/n*, where *n* is the atomic density. At energies of several hundred keV/u and higher, energy loss is dominated by the energy transfer due to excitation of electrons in binary collisions with the penetrating ion in an otherwise weakly perturbed solid. At lower energies the energy loss process becomes more complex. The projectile may bind electrons, therefore charge state effects must be considered. Consequently, screening of the projectile charge by both projectile and target electrons becomes of importance. With a decrease in ion energy, and consequently of the maximum energy transfer in a binary ion-electron collision, details of the electronic structure of the solid get highly relevant. For the interaction of nuclei multiple scattering effects must be taken into account due to the increase of scattering cross sections^4–6^.

To model inelastic excitations of a solid, a free electron gas (FEG) model can be employed. This model is simple but powerful and has shown to be capable of giving accurate numeric predictions of the electronic stopping power^7^. The electronic stopping power S_e_ for ion velocities *v* <= *v*_*F*_ (with *v*_*F*_ the Fermi speed) in a target described as a FEG with an effective density *n*_*e*_ (usually expressed by the Wigner-Seitz radius ${r}_{S}={(\frac{3}{4\pi {n}_{e}})}^{\frac{1}{3}}$) is expected to be proportional to *v*, *S*e = *Q(Z*_*1*_, *r*_*S*_*)v*, with *Z*_*1*_ the projectile atomic number and *Q* the friction coefficient^8^. Density functional theory (DFT) has been extensively used to calculate the stopping power for a FEG with an appropriate density representing the intended material^9,10^. These calculations performed for different projectiles predict oscillations in the electronic stopping power as a function of *Z*_*1*_^11–13^ with a maximum at *Z*_*1*_ ~ 6 and a minimum at *Z*_*1*_ ~ 11 (depending on the effective density) due to the projectile electronic structure. Echenique *et al*.^11^. studied the friction coefficients for ions with *Z*_*1*_ from 1 to 18 for different FEG densities. Their results show that the positions of the maximum and minimum shift to higher values of *Z*_*1*_ with decreasing *r*_*S*_. Such *Z*_*1*_ oscillations have been observed experimentally^14–18^ and for some materials discrepancies with DFT calculations are observed^18^.

In this work we investigate the electronic stopping power of titanium nitride for different ions at energies corresponding to ion velocities below 1 atomic unit (a.u.). We deduced the experimental electronic SCS (*ε*_*e*_) for H, He, B, N, Ne and Al in TiN by backscattering spectrometry using time-of-flight medium-energy ion scattering (TOF-MEIS) and Monte Carlo simulations. Thin films of TiN grown on silicon with a thin layer of W at the interface, were used in our experiments. The results were compared with SRIM^19^ results and with nonlinear DFT calculation for a FEG according to Nagy *et al*.^10^.

## Methods

For the TOF-MEIS based energy loss investigations we used thin polycrystalline TiN films on silicon, with a W layer of 1 nm nominal thickness at the interface. The sample was prepared by sputtering in a Kurt J. Lesker CMS-18 deposition system by the Department of Engineering Sciences at Uppsala University. The sample was characterized by Rutherford backscattering spectrometry (RBS) and time-of-flight elastic recoil detection analysis (TOF-ERDA) with ion beams of 2 MeV ^4^He^+^ and 36 MeV ^127^I^8+^, respectively. The beams were provided by a 5 MV 15SDH-2 tandem accelerator at the Tandem Laboratory at Uppsala University. To obtain the areal thickness of the TiN films, RBS measurements were performed with off-axis incident beam using a series of small random tilt angles on the sample around an equilibrium position, to minimize channelling effects in particular due to the Si substrate. The stoichiometry of the film was quantified and possible contaminations were identified by TOF-ERDA experiments^20,21^, with incident and detection angles of 67.5° with respect to the surface normal. The resulting thickness of the TiN films is 17.5 ± 0.6 nm (assuming a TiN density of 5.43 g/cm^3^) with a stoichiometry Ti:N = 1 ± 0.08. The thickness of the W layer is 1.3 ± 0.1 nm. Minor oxygen impurities (below 5%) were observed only on the surface of the sample.

**Figure 1:**
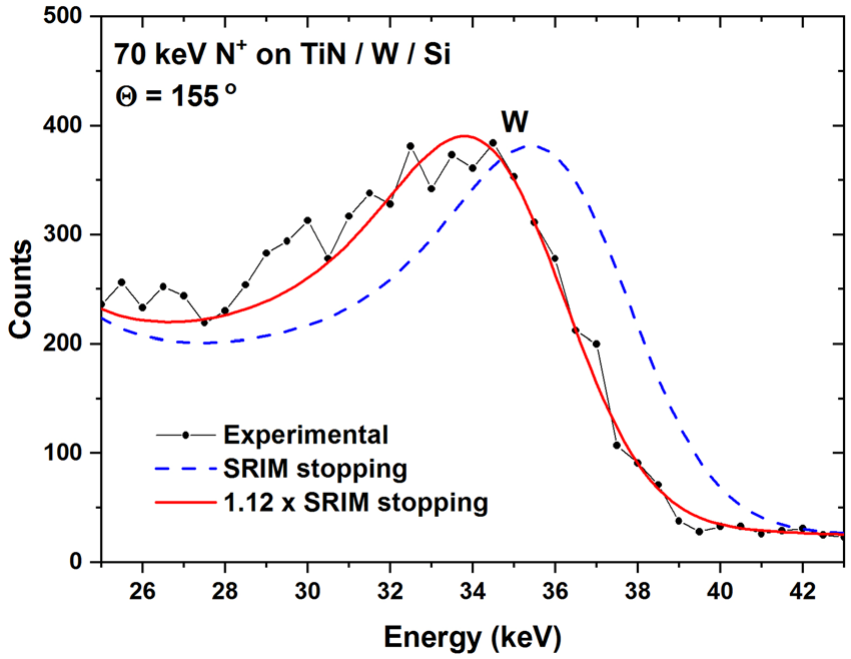
Energy converted TOF-MEIS spectrum for 70 keV N^+^ (black dots) scattered from a sample consisting of a titanium nitride film on silicon with a δ-layer of tungsten at the interface. By TRBS simulations, the correct electronic stopping power is obtained from the shift of the W peak position due to energy loss in the TiN film (solid red line). The dashed line (blue) shows the simulation using electronic stopping by SRIM.

To obtain energy loss data, we performed TOF-MEIS experiments at the setup at the Ångström Laboratory in Uppsala^22^, using H, He, B, N, Ne and Al as projectiles with energies in the range of 3 to 140 keV/u. The beams for TOF-MEIS were provided by a Danfysik 350 kV ion implanter. Backscattered particles are detected by a large area position sensitive MCP detector, with a solid angle *Ω* > 0.1 sr. Only particles detected at scattering angles between 153° and 157° were selected for evaluation, to avoid effects of geometrical straggling. For H and He projectiles, electronic stopping data are obtained from the width of the spectrum signal corresponding to scattering from Ti. For heavier ions, the position of the peak corresponding to scattering from the W δ-layer is used, as shown in Fig. 1 for a 70 keV N^+^ beam. To account for the influence of multiple scattering and associated nuclear losses, evaluations were performed by Monte Carlo simulation of the experimental data employing the TRIM for Backscattering code (TRBS)^23^. Note that in the simulations it is also possible to tune the employed potential^24^. Previous investigations have, however, shown only a very minor impact on the observed spectrum width, even for the case of Ne ions in a heavy matrix such as Au^25^. The experimental stopping power is now obtained from the TRBS simulation by applying a correction factor to the employed electronic stopping power until a best fit to the experiment is achieved. Note that electronic stopping data for H, He and Ne have been reported by Sortica *et al*.^26^. The systematic uncertainty of the deduced electronic stopping from the film thickness calibration by RBS is estimated to be ~4% due to possible residual channelling in the substrate, accuracy of the employed stopping power for MeV He in Si and experimental statistics. In the MEIS experiments, no evidence for channelling of ions backscattered from TiN could be observed. The statistical uncertainty due to the fitting procedure using TRBS is estimated to be below 2%. Considering other possible uncertainties due to film stoichiometry, binning effects, etc., the accumulated uncertainty of our deduced electronic stopping cross sections should be below 7%.

## Results and Discussions

**Figure 2:**
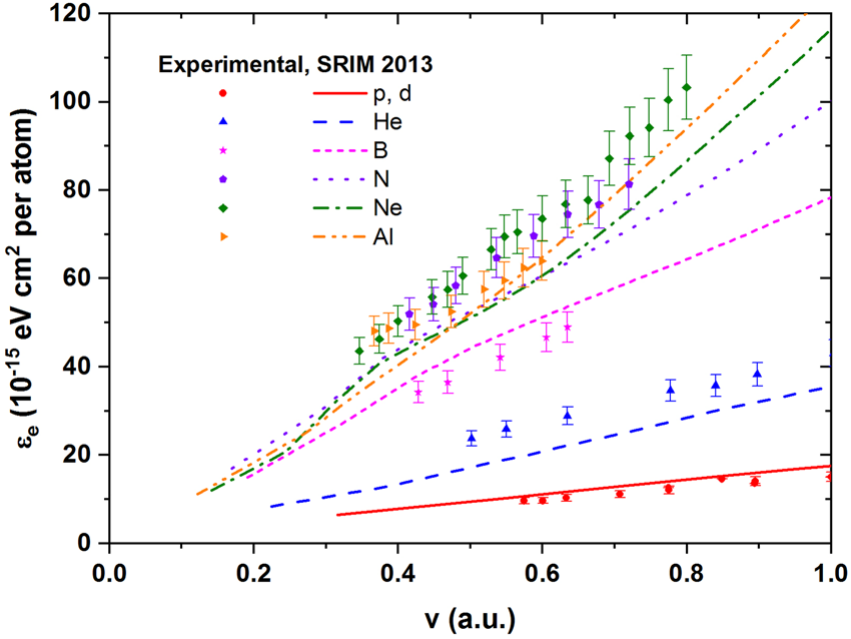
Experimental electronic stopping cross sections *ε*_*e*_ for H, He, B, N, Ne and Al ions in TiN (full symbols). Also shown are the corresponding SRIM data.

Figure 2 presents experimental results for the stopping cross section *ε*_*e*_ for all investigated projectiles together with the corresponding SRIM results for ion velocities *v* up to 1 a.u.. For H and Ne projectiles, *ε*_*e*_ is observed to be proportional to *v*. For He, B and N, *ε*_*e*_ exhibits a linear dependence on *v* extrapolating to a positive offset at zero velocity. For Al projectiles, at the lowest energies a clear non-linear dependence of *ε*_*e*_ on the ion velocity is observed. Note, however, that experiments for heavier ions at lowest energies have highest systematic and statistical uncertainty due to the evaluation procedure being more affected from nuclear losses.

**Figure 3:**
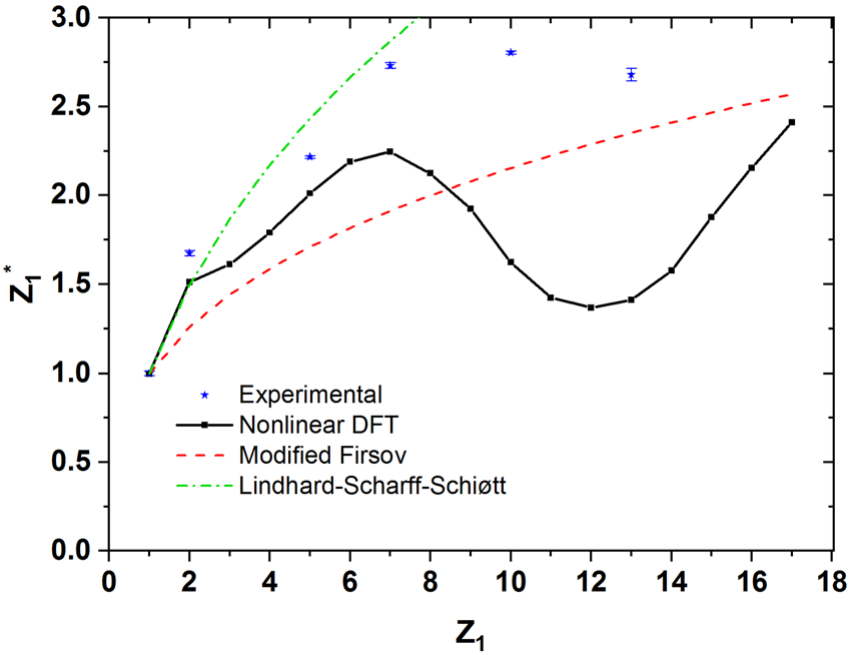
Effective charge *Z*_1_* as a function of *Z*_*1*_ as calculated for a free-electron gas with electron density equivalent to TiN (black dots). Blue asterisks show our results as deduced from the observed stopping cross sections *ε*_*e*_ for H, He, B, N, Ne, and Al. For details see text.

The deduced values of the stopping cross sections are subsequently compared to nonlinear DFT calculations for a FEG according to Nagy *et al*.^10^. The density parameter *r*_*S*_ = 1*.6*1 *a.u*. for the FEG (which corresponds to ~7 electrons per molecule) was obtained from the experimental plasmon energy of TiN^27^. On this basis, for protons, excellent agreement with DFT predictions has been found^26^. Note, that the electronic structure of TiN, although being a metallic compound, is significantly different from a FEG^28^. Also, it has been recently shown, that for heavier early transition and rare-earth elements the DFT-model has failed as unreasonably high electron densities would have been required^29^. For all projectiles heavier than hydrogen, DFT calculations systematically underestimate the experimental data. To present the electronic stopping cross section as a function of the projectile atomic species and to allow for a clear comparison with theory, our results are expressed in terms of ${Z}_{1}^{\ast }={(\frac{dE}{dx}({Z}_{1})/\frac{dE}{dx}(Z=1))}^{1/2}$, defined as the effective charge by Echenique *et al*.^11^. For each *Z*_*1*_, *ε*_*e*_ is obtained from the corresponding plot shown on Fig. 2, assuming it to be proportional to *v* for *v* < 1 a.u. Figure 3 shows the calculated *Z*_1_^*^ and experimental values for H, He, B, N and Al as a function of *Z*_*1*_. Oscillations in *Z*_1_^*^ are present in the calculations due to screening effects and the atomic structure of the projectile^11^. To allow for an additional comparison with the model by Lindhard and Scharff^30^ as well as the modified Firsov-formula^31^ we have plotted their respective predictions as dashed and dash-dotted lines respectively. As both models ignore the shell structure of atoms and thus by definition are incapable of reproducing possible *Z*_*1*_-oscillations in dE/dx, both predict a monotonic increase in *Z*_1_^*^. Note, that also the observed slopes are significantly different from the experimental observations. When comparing with DFT, as already mentioned, except for protons, the calculated stopping power is always found lower than the experimental data, with increasing discrepancy for increasing *Z*_*1*_. In contrast to predictions, our data exhibit a broad maximum close to the calculated minimum, which has been attributed to atomic numbers corresponding to full atomic shells of the projectile. However, results obtained in grazing surface scattering on LiF confirm the predicted *Z*_*1*_ oscillations very well^18^. This qualitative difference in the *Z*_*1*_-dependence of electronic stopping of slow ions in surface scattering and in a solid points towards an additional energy loss mechanism in the latter case. Such a process should have high efficiency for ions with atomic numbers close to full atomic shells and is expected to occur for comparably small impact parameters. We suggest electron promotion in atomic collisions between dressed atoms as explanation. In collisions of both, charged or neutral projectiles with a target atom, promotion of electrons due to Pauli repulsion will provide an efficient mechanism of ionization^32^. In parallel, a formation of molecular orbitals at short interaction distances with an associated modification of the total energy of the electronic system can be expected^33^. Both processes can lead to electronic energy loss independent from electron-hole pair excitation in a Coulomb collision, and, therefore, increase electronic stopping.

The proposed interpretation of the *Z*_*1*_-dependence of electronic stopping is supported by the observed deviations from velocity proportionality for *ε* for some of the projectiles. The relevance of atomic collisions is corroborated by the fact that inner shell excitation of the target material is observed even for low energy He projectiles^34,35^. As a consequence, repeated charge-exchange cycles and internal excitations of the projectile will occur and contribute to the electronic stopping power, as has been observed for He and Ne ions at energies significantly below 10 keV^36,37^. A possible influence of these processes on the velocity scaling of the electronic energy loss observed for He ions in Al and Au has been reported^38,39^. Note that electron transfer in atomic collisions can lead to substantial energy transfer that clearly exceeds the maximum energy transfer in a binary ion-electron collision^35^.

## Summary

In this work, electronic stopping cross sections for B, N and Al in TiN have been measured. Experimental results, including H, He and Ne from Sortica *et al*.^26^ are compared with predictions from theory, in particular non-linear DFT calculations for a FEG. For protons, DFT-calculations perfectly reproduce the experimental data^26^, which indicates that in this case electronic stopping is dominated by direct excitation of conduction electrons of TiN in binary ion-electron collisions. For heavier ions, the discrepancy between predictions by theory and experimental data is found to increase with the projectile atomic number. For these ions, direct electron-hole pair excitation in binary collisions is apparently insufficient to explain the observed increase in the stopping cross sections. More complex dynamic energy transfer processes are expected to be responsible for the apparent discrepancy between static theory and experiment. Time dependent DFT calculations, including contributions of inner shells and the projectile electronic structure may lead to a better understanding of energy-loss processes of ions in TiN.

## Data Availability

All data generated during this study are available from the corresponding author on reasonable request and are publicly available on https://www-nds.iaea.org/stopping.
